# A Phenomenological Synapse Model for Asynchronous Neurotransmitter Release

**DOI:** 10.3389/fncom.2015.00153

**Published:** 2016-01-14

**Authors:** Tao Wang, Luping Yin, Xiaolong Zou, Yousheng Shu, Malte J. Rasch, Si Wu

**Affiliations:** ^1^State Key Laboratory of Cognitive Neuroscience and Learning and IDG/McGovern Institute for Brain Research, Beijing Normal UniversityBeijing, China; ^2^Institute of Neuroscience and State Key Laboratory of Neuroscience, Shanghai Institutes for Biological Sciences, Chinese Academy of Sciences and University of Chinese Academy of SciencesShanghai, China

**Keywords:** asynchronous release, synchronous release, neurotransmitter, synaptic model, short-term plasticity, Ca^2+^ concentration, stochasticity

## Abstract

Neurons communicate with each other via synapses. Action potentials cause release of neurotransmitters at the axon terminal. Typically, this neurotransmitter release is tightly time-locked to the arrival of an action potential and is thus called synchronous release. However, neurotransmitter release is stochastic and the rate of release of small quanta of neurotransmitters can be considerably elevated even long after the ceasing of spiking activity, leading to asynchronous release of neurotransmitters. Such asynchronous release varies for tissue and neuron types and has been shown recently to be pronounced in fast-spiking neurons. Notably, it was found that asynchronous release is enhanced in human epileptic tissue implicating a possibly important role in generating abnormal neural activity. Current neural network models for simulating and studying neural activity virtually only consider synchronous release and ignore asynchronous transmitter release. Here, we develop a phenomenological model for asynchronous neurotransmitter release, which, on one hand, captures the fundamental features of the asynchronous release process, and, on the other hand, is simple enough to be incorporated in large-size network simulations. Our proposed model is based on the well-known equations for short-term dynamical synaptic interactions and includes an additional stochastic term for modeling asynchronous release. We use experimental data obtained from inhibitory fast-spiking synapses of human epileptic tissue to fit the model parameters, and demonstrate that our model reproduces the characteristics of realistic asynchronous transmitter release.

## 1. Introduction

Most neurons are connected with chemical synapses that release neurotransmitters to communicate. A synapse connects the axon of a presynaptic neuron with the dendrite of a postsynaptic neuron and has complex physiological structure including several functional sub-compartments (Sudhof, 2004). In particular, neurotransmitters are stored in vesicles located at the axon terminal, called the active zone (Paradiso et al., 2007). Upon arrival of an axonic action potential, the voltage-gated Ca^2+^ channels in the active zone open, leading to the influx of Ca^2+^-ions that bind to Ca^2+^ sensors on the vesicles. When sufficiently many Ca^2+^-ions are bound, sensors activate and the fusion of the vesicles with the membrane is initiated. Consequently, the neurotransmitters stored inside the vesicles are released to the outside of the axon terminal. They then diffuse to the postsynaptic site, bind to membrane receptors, and initiate a sequence of biochemical reactions in the postsynaptic neurons, e.g., activate ion channels that change the membrane voltage possible resulting in firing activity of the post synaptic neuron (Sudhof, 2004).

The release of neurotransmitters is generally time-locked to the arrival of an action potential, so that the former occurs immediately after the latter; a process called synchronous release. However, in some types of neurons and in certain circumstances, significant amount of neurotransmitters can be released asynchronously to the arrival of action potentials (Goda and Stevens, 1994). Asynchronous release of neurotransmitters is stochastic and may occur and persist long after the last arrival of an action potential (Chapman, 2008).

Although synchronous release predominates in most synapses, asynchronous release can be strong in certain types of chemical synapses, in particular, when the presynaptic neuron fires at high frequency (Hefft and Jonas, 2005; Daw et al., 2009). Notably, it was found that in fast-spiking interneurons in human epileptic tissues, asynchronous GABA release increases significantly compared to control tissues (Jiang et al., 2012), indicating that asynchronous neurotransmitter release may play an important role in generating or compensating for abnormal neural activities. Although it has been speculated in several studies that asynchronous release may contribute to enhancing neuronal inhibitory interactions (Medrihan et al., 2014) and desynchronizing network responses (Manseau et al., 2010), the exact role of asynchronous release in brain function remains largely unknown and unexplored.

One reason for the lack of understanding of the effect of asynchronous release onto neural activity is that virtually all modeling studies of brain functions only include synchronous neurotransmitter release and neglect the existence of asynchronous release. While many phenomenological models of various degrees of complexities exist for modeling synchronous release—from simple exponential decays (Destexhe et al., 1994; Dayan and Abbott, 2003) and delayed alpha functions (Rall, 1967) to more-variable models that include short-term plasticity (Markram and Tsodyks, 1996; Tsodyks and Wu, 2013)—a simple phenomenological model of the effect of asynchronous release onto the membrane voltage of the postsynaptic neurons does not exist. Thus, the computational neuroscience toolbox currently does not provide a simple mathematical framework to describe the dynamics of asynchronous release that could be easily included in large-scale neural network models.

Therefore, to enable the study of the role of asynchronous release in brain functions by using a neural network modeling approach, we here develop a simple phenomenological model for synaptic release that includes both, synchronous and asynchronous transmitter release. We base our description on a common synaptic model for short-term synaptic plasticity (STP), that phenomenologically describes the dynamics of the synchronous release (Loebel et al., 2009; Tsodyks and Wu, 2013). We thus call our model the synchronous-asynchronous release STP model (short: SAR model). By comparison to experimental data, we show that the SAR model, despite being simple enough to support large-size network simulation, nevertheless captures the essential features of both transmitter release processes.

## 2. Results

### 2.1. Derivation of the SAR model

**Figure 1:**
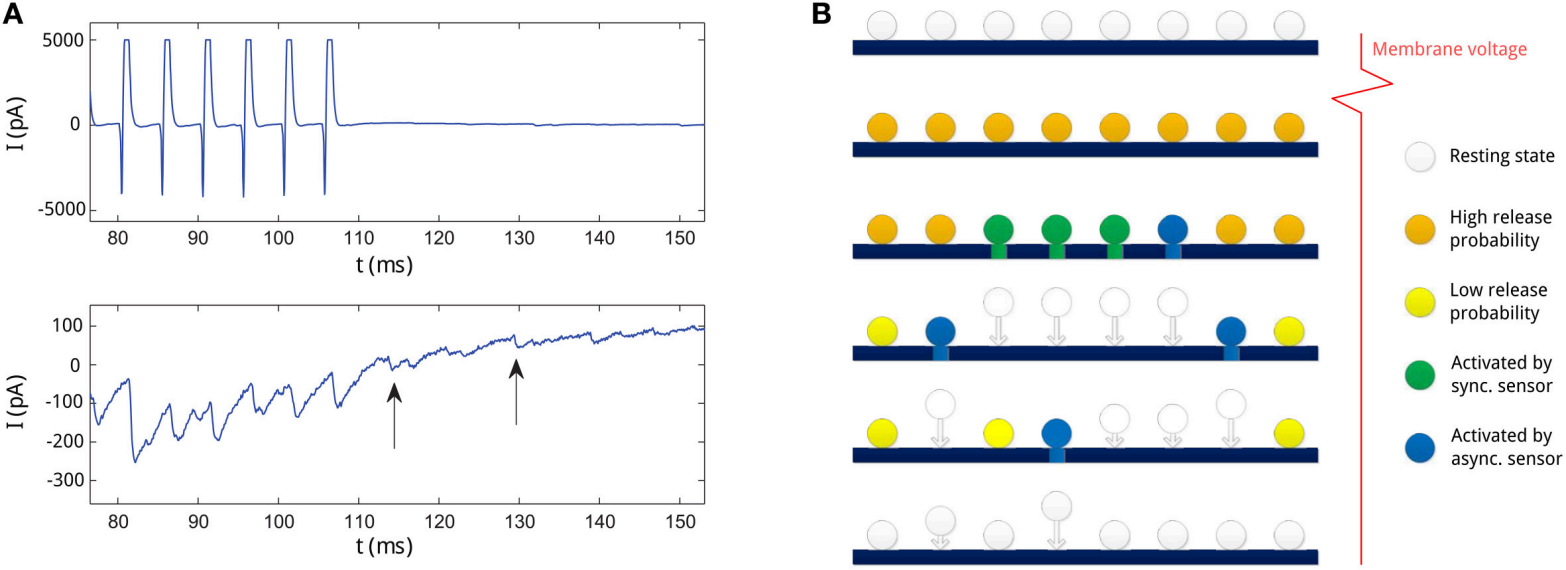
**(A)** An example of asynchronous release recorded at a presynaptic neuron from Jiang et al. (2012). Upper panel: the membrane current at the presynaptic neuron. The current pulses represent action potentials at the presynaptic neuron. Lower panel: the IPSC at the postsynaptic neuron. Two asynchronous release events long after action potentials are indicated with arrows. **(B)** Illustration of the neurotransmitter release process in the SAR model for consecutive times (from upper to lower row) in the active zone. First row: at resting state, vesicles dock at the membrane in the active zone, the amount of Ca^2+^ bound to the Ca^2+^ sensors in the vesicles in low (indicated color by the white color). Second row: after an action potential, Ca^2+^ binds to the sensors on the vesicles (orange color), and vesicles have high release probability. Third row: soon after the action potential, some vesicles (marked green and blue) are activated by the Ca^2+^ sensors and start to release transmitters. Activation of vesicles can stem from the synchronous release (green) or the asynchronous release (blue) process competing for vesicles. Forth row: Vesicles released need to be replenished and newly recruited to the active zone (indicated with arrows). In this time, they are not available for release. Due to Ca^2+^ clearance process in the neuron, remaining available vesicles decrease in release probability (yellow). However, some vesicles are still activated by the asynchronous release sensors and start to release transmitters (blue). Some other vesicles are being replenished at the same time. Fifth row: as the release probability decreases, less vesicles are activated by the asynchronous release process. Last row: as Ca^2+^ is cleared, all vesicles return to the resting state and release is stopped. Neurotransmitter release will only be newly initiated by spiking activity.

We first show an example measurement of experimentally observed asynchronous transmitter release. Figure 1A shows presynaptically and postsynaptically measured currents measured at the synapse from a fast-spiking interneuron to a pyramidal neuron in a human epileptic tissue (same data as described in Jiang et al., 2012). Note that large inhibitory postsynaptic currents (IPSC) are coupled with the spikes of the presynaptic neurons (in the time range of 80–110 ms), indicating synchronous release. However, IPSC are variable and stochastic release events can be seen even after the spiking activity (indicated with arrows), which we refer to as asynchronous release.

To develop an adequate synaptic model describing both asynchronous and synchronous synaptic activity, we first review a well-known model describing synchronous release, which we will later extend to include asynchronous release as well.

#### 2.1.1. STP model for synchronous release

The classical phenomenological model for describing the short-term dynamics of synaptic currents of the synchronous neurotransmitter release was introduced by Markram and Tsodyks (1996). This model postulates a pool of releasable vesicle and a dynamically changing release probability (e.g., depending on the changing Ca^2+^ concentration). Let *u* denote the release probability and *x* the amount of neurotransmitters contained in the readily releasable vesicles. Each presynaptic spike induces the current fraction *u*(*t*) of vesicles to release all their neurotransmitters, which subsequently causes a postsynaptic voltage change.

The dynamics of *u* and *x* are given by Tsodyks and Wu (2013),

(1) $$\begin{array}{lll} \frac{du\left(t\right)}{dt} & =-\frac{u\left(t\right)}{\tau_{f}}+U\left[1-u\left(t\right)\right]\underset{m}{\sum }\delta \left(t-t_{m}\right),\; & \; \end{array}$$

(2) $$\begin{array}{lll} \frac{dx\left(t\right)}{dt} & =\frac{X_{F}-x\left(t\right)}{\tau_{d}}-q\left(t\right),\; & \; \end{array}$$

(3) $$\begin{array}{lll} q\left(t\right) & =u\left(t_{+}\right)x\left(t_{-}\right)\underset{m}{\sum }\delta \left(t-t_{m}\right),\; & \; \end{array}$$

where *t*_*m*_ denotes the arriving moment of the *m*th spike, and δ(·) is the Dirac delta function. Note that in comparison to the original model in Markram and Tsodyks (1996), we have here for convenience changed the definition of the variable *x* to be the amount of neurotransmitter available rather than the fraction of neurotransmitters available. Further, *U* denotes the increment of *u* due to the arrival of an action potential. The time constant τ_*f*_ controls the rate of the decay of *u*, and τ_*d*_ controls the rate of replenishment of vesicles. *X*_*F*_ is the total amount of neurotransmitters in all vesicles at the active zone, when all vesicles are replenished. The notion *t*_+_ and *t*_−_ indicates, respectively, the moments of just after and before the arrival of an action potential. *q*(*t*) is the rate of neurotransmitter release at a given time *t*. According with the synchronous release process, this neurotransmitters release here is time-locked to the arrival of a presynaptic spike (delta function in Equation 3).

Finally, a simple way to describe the induced postsynaptic membrane current *I*_syn_ due to the released neurotransmitters is Destexhe et al. (1994), Dayan and Abbott (2003)

(4) $$\begin{array}{lll} I_{\text{syn}}\left(t\right) & =g\left(t\right)\left(v\left(t\right)-E_{\text{syn}}\right),\; & \; \end{array}$$

(5) $$\begin{array}{lll} \frac{dg\left(t\right)}{dt} & =-\frac{g\left(t\right)}{\tau_{\text{syn}}}+wq\left(t-D\right),\; & \; \end{array}$$

where *v*(*t*) is the membrane potential, *g*(*t*) is the synaptic conductance at the postsynaptic site, *E*_syn_ the reversal potential of the synapse, τ_syn_ the time constant of the synaptic ion-channels, *w* the synaptic efficacy produced by one unit of neurotransmitter, and *D* the time delay due to the signal transmission.

#### 2.1.2. Extension of the STP model to include asynchronous release

We extend the above STP model to include the asynchronous release process. The simple dynamics of the synchronous release probability in the STP model provides an adequate framework for modeling the asynchronous release since it allows for implementing a slowly changing asynchronous release rate that is modulated by firing activity as observed in experiments (Wen et al., 2010; Jiang et al., 2012).

Molecular biological studies found that synchronous and asynchronous neurotransmitter release are mediated via different Ca^2+^ sensors (Sun et al., 2007; Xu et al., 2009; Wen et al., 2010; Bacaj et al., 2013) that have distinct association and dissociation rates with Ca^2+^. We therefore use two variables, *u*_sr_ and *u*_ar_, to denote their corresponding release probabilities, that is, *u*_sr_ denotes the release probability due to synchronous release during spiking events, and *u*_ar_ the release probability due to the asynchronous release per unit time.

In the SAR model, we assume that for both release probabilities the qualitative dynamics remain identical to the STP model (Equation 1) although parameters are different in general. We thus write

(6) $$\begin{array}{lll} \frac{du_{\text{sr}}\left(t\right)}{dt} & =-\frac{u_{\text{sr}}\left(t\right)}{\tau_{\text{sr}}}+U_{\text{sr}}\left[1-u_{\text{sr}}\left(t\right)\right]\underset{m}{\sum }\delta \left(t-t_{m}\right),\; & \; \end{array}$$

(7) $$\begin{array}{lll} \frac{du_{\text{ar}}\left(t\right)}{dt} & =-\frac{u_{\text{ar}}\left(t\right)}{\tau_{\text{ar}}}+U_{\text{ar}}\left[U_{\text{max}}-u_{\text{ar}}\left(t\right)\right]\underset{m}{\sum }\delta \left(t-t_{m}\right),\; & \; \end{array}$$

where *U*_sr_ and *U*_ar_ denote, respectively, the increments of release probability induced by an action potential via the synchronous and asynchronous release pathways, and τ_sr_ and τ_ar_ denote, respectively, the dissociation rates of Ca^2+^ from the two kinds of sensors. Note that technically, since the synchronous release is locked to instantaneous events while the asynchronous release process is an ongoing process, the *u*_sr_ is a release probability whereas *u*_ar_ is a probability rate. We thus add an additional parameter, *U*_max_, representing the saturation level of the facilitation for asynchronous release probability rate.

Experimental studies further suggest that fluctuations of synchronous and asynchronous releases are anti-correlated, implying that they are competing for the same pool of vesicles (Wen et al., 2010). We therefore use a single variable *x* as in Equation (2) to denote the amount of readily available neurotransmitters.

As before, the synchronous release of neurotransmitters is time-locked to the arrival of an action potential, and rate of release is given by

(8) $$\begin{array}{l} q_{\text{sr}}\left(t\right)=u_{\text{sr}}\left(t_{+}\right)x\left(t_{-}\right)\underset{m}{\sum }\delta \left(t-t_{m}\right). \end{array}$$

The asynchronous release of neurotransmitters is, on the other hand, highly stochastic. This stochasticity can be modeled as a binomial process (Del Castillo and Katz, 1954). A random variable *n* is said to be binomial, *n* ~ B(*M, p*), when it follows the distribution $P(n=k|M,p)=\left(\begin{array}{c} N\\ k \end{array}\right)p^{k}{\left(1-p\right)}^{N-k}$, with *k* ∈ {0, …, *M*} and *p* the probability of a single event.

Suppose that *x*_0_ is the amount of neurotransmitters contained in a vesicle, called the quantum size. Thus, the maximal number of readily releasable vesicles at time *t* is therefore ~ ⌊*x*(*t*)/*x*_0_⌋, with the symbol ⌊·⌋ denoting taking the nearest integer number from below. Further, in a small time interval [*t, t* + *dt*), the release probability of a single vesicles due to asynchronous release is *u*_ar_(*t*)*dt*. Therefore, when assuming that the number of asynchronous release events is binomial distributed, it is given by a binomial random variable *n*(*t*) that follows the binomial distribution B(⌊*x*(*t*)/*x*_0_⌋, *u*_ar_(*t*)*dt*). Thus, the amount of neurotransmitters released in the interval [*t, t* + *dt*) is given by

(9) $$\begin{array}{l} q_{\text{ar}}\left(t\right)dt=x_{0}n_{\text{ar}}\left(t\right), \end{array}$$

where *n*_ar_(*t*) is the binomial random variable defined above.

When calculating the joint rate of synchronous and asynchronous releases, in principle we have to correct for the case that some active vesicle were released due to the asynchronous release and are thus not available anymore for the synchronous release during spiking activity. However, since spikes are instantaneous events, and in practice the time interval is very small, the overlap of the two processes can be neglected. Thus, the overall neurotransmitter release rate at *t* is given by

(10) $$\begin{array}{l} q\left(t\right)=q_{\text{ar}}\left(t\right)+q_{\text{sr}}\left(t\right). \end{array}$$

The Equations (2, 6, 7, 10) jointly define the synaptic dynamics of a release model, that includes stochastic asynchronous besides a deterministic synchronous release process.

#### 2.1.3. Including stochastic synchronous release in the SAR model

While in the previous section the synchronous release is based on the deterministic STP model, in reality the synchronous release process is stochastic (Loebel et al., 2009). To arrive at our final SAR-model, we replace the deterministic STP model by a stochastic version, as suggested previously (Loebel et al., 2009). For that, we add two stochastic processes, one to describe the number of released vesicles by the synchronous release, *n*_sr_(*t*), and the other describing the number of replenished vesicles, *r*(*t*). These variables can be similarly modeled as binomial processes as shown previously. For the number of released vesicles triggered by the spikes by the synchronous release, it is thus

(11) $$\begin{array}{l} n_{\text{sr}}\left(t\right)\sim B\left(N\left(t\right),u_{\text{sr}}\left(t_{+}\right)\right), \end{array}$$

where *N*(*t*) is the number of available vesicle resources, that is *N*(*t*) = ⌊*x*(*t*)/*x*_0_⌋ in the notation of the last subsubsection and the *u*_sr_ is the release probability given by Equation (6). Second, for the number of replenished vesicles at time *t* it is

(12) $$\begin{array}{l} r\left(t\right)\sim B\left(N_{F}-N\left(t\right),dt∕\tau_{d}\right), \end{array}$$

implementing a stochastic depression process analogous to Equation (2), with parameter *N*_*F*_ = ⌊*X_F_*/*x*_0_⌋. Taken together, the number of available vesicles at time *t* in the common pool, *N*(*t*), changes according to the released and replenished vesicles at time *t*, thus

(13) $$\begin{array}{l} dN\left(t\right)=r\left(t\right)-n_{\text{ar}}\left(t\right)-n_{\text{sr}}\left(t\right)\underset{m}{\sum }\delta \left(t-t_{m}\right)dt. \end{array}$$

Finally, the synchronous release rate changes to

(14) $$\begin{array}{l} q_{\text{sr}}\left(t\right)=x_{0}n_{\text{sr}}\left(t\right)\underset{m}{\sum }\delta \left(t-t_{m}\right) \end{array}$$

**Figure 2:**
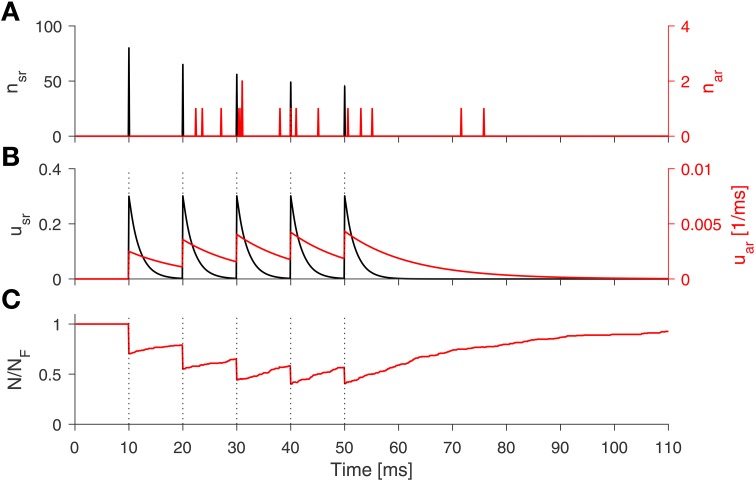
**Numerical simulation of the SAR-model**. The spike train consist of 5 spikes (100 Hz, black dotted lines). **(A)** The number of released vesicle according to the stochastic processes for the synchronous (black line) and asynchronous release (red line). **(B)** Dynamics of the release probability of synchronous (black) and asynchronous release (red line). **(C)** Fraction of available vesicles in the pool. *Parameters:* τ_sr_ = 2 ms, *U*_sr_ = 0.3, τ_ar_ = 12 ms, *U*_ar_ = 0.005, τ_*d*_ = 30 ms, *U*_max_ = 0.5, and *N*_*F*_ = 271.

These equations complete our SAR model, which is illustrated in Figure 1B. The equations jointly defining the synaptic dynamics of the SAR model, which includes both stochastic synchronous and asynchronous neurotransmitter release, are summarized in the Algorithm 1. A numerical example simulation of the SAR model is shown in Figure 2.

**Algorithm 1:** **Synchronous-Asynchronous Release STP Model**.

Sync. release probability:	$\begin{array}{l} \frac{\text{d}u_{\text{sr}}\left(t\right)}{\text{d}t}=-\frac{u_{\text{sr}}\left(t\right)}{\tau_{\text{sr}}}+U_{\text{sr}}\left[1-u_{\text{sr}}\left(t\right)\right]\\ \sum_{m}\delta \left(t-t_{m}\right) \end{array}$
Async. rel. probability rate:	$\begin{array}{l} \frac{\text{d}u_{\text{ar}}\left(t\right)}{\text{d}t}=-\frac{u_{\text{ar}}\left(t\right)}{\tau_{\text{ar}}}\text{​ }+\text{ ​}U_{\text{ar}}\left[U_{\text{max}}\text{​}-\text{ ​}u_{\text{ar}}\left(t\right)\right]\\ \sum_{m}\delta \left(t-t_{m}\right) \end{array}$
Sync. release rate:	$q_{\text{sr}}(t)=x_{0}n_{\text{sr}}(t)\sum_{m}\delta \left(t-t_{m}\right)$
Async. release rate:	$q_{\text{ar}}(t)\text{d}t=x_{0}n_{\text{ar}}(t)$
Available vesicle number:	$\begin{array}{l} \text{d}N(t)=r(t)-n_{\text{ar}}(t)-n_{\text{sr}}(t)\\ \sum_{m}\delta \left(t-t_{m}\right)\text{d}t \end{array}$
Vesicles replenishment:	$r(t)\sim B\left(N_{F}-N(t),\text{ d}t/\tau_{d}\right)$
Sync. released vesicles:	$n_{\text{sr}}(t)\sim B\left(N(t),\;u_{\text{sr}}(t_{+})\right)$
Async. released vesicles:	$n_{\text{ar}}(t)\sim B\left(N(t),\;u_{\text{ar}}(t)\text{d}t\right)$

### 2.2. Estimation of the parameters of the SAR model

To apply the SAR model in network simulations in practice, we need to find a working set of its free parameters, which are τ_sr_, τ_ar_, *U*_sr_, *U*_ar_, τ_*d*_, *N*_*F*_, *U*_max_, and *x*_0_. In general, these parameters may vary in different cortical areas and for different types of synapses, analogous to the parameters of the STP model for synchronous release (Silberberg et al., 2005). Note that the SAR model includes short-term plasticity of the synchronous release, so that facilitating and depressing synapse dynamics can be expressed with the model in addition to dynamics of the asynchronous release.

In the following, we develop a method which fits the free parameters according to experimental data. We use experimental data from a synapse connecting a fast-spiking neuron to a pyramidal neuron as an example case (Jiang et al., 2012). We proceed in two steps. First, the time course of the release rate has to be estimated from the raw IPSC traces. Second, we use the release rate time course to estimate parameters of the model by probabilistic inference.

#### 2.2.1. Extracting the neurotransmitter release rate from the recorded IPSC

We first extract the release rate of neurotransmitters from the inhibitory postsynaptic currents (IPSCs) recorded at the postsynaptic pyramidal neuron.

Let *I*_syn_ denote the synaptic current and *g*(*t*) the synaptic conductance at the postsynaptic neuron. We use Equations (4) and (5) to describe the synaptic currents induced by GABA receptors, and have

(15) $$\begin{array}{lll} I_{\text{syn}}\left(t\right) & =g\left(t\right)\left(v-E_{\text{GABA}}\right),\; & \; \end{array}$$

(16) $$\begin{array}{lll} \frac{dg\left(t\right)}{dt} & =-\frac{g\left(t\right)}{\tau_{\text{GABA}}}+wq\left(t-D\right),\; & \; \end{array}$$

with analogous variables as in Equations (4) and (5).

The experimental data was recorded using voltage-clamp, the membrane voltage *v* was fixed and *E*_*GABA*_ was stable when the neuron was at equilibrium, therefore *A* ≡ *w*(*v* − *E*_*GABA*_) is a constant (Jiang et al., 2012). With this condition, the above equations are solved for *I*_syn_(*t*),

(17) $$\begin{array}{l} I_{\text{syn}}(t)=\int_{-\infty }^{t}e^{-\frac{t-t\prime }{\tau_{\text{GABA}}}}Aq(t\prime -D)\text{ d}t\prime . \end{array}$$

**Figure 3:**
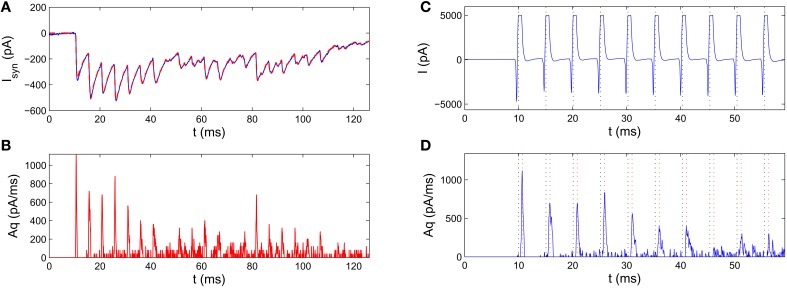
**Pre-processing of the raw IPSC recordings for estimating parameters of the SAR model**. The experimental data was recorded from a synapse connecting a fast-spiking neuron to a pyramidal neuron in a human epileptic tissue (Jiang et al., 2012). **(A)** Blue curve: an example IPSC trace. Red dashed curve: the reconstructed IPSC trace based on the extracted release rate (from **B**). The two curves agree very well with each other. **(B)** Extracting release rate from experimentally measured IPSC traces. The scaled release rate $\tilde{q}=Aq$ extracted from the IPSC in A by the deconvolution method. **(C,D)** Estimation of the transmission delay by comparison of the time of spikes (black dotted lines) in the pre-synaptic voltage trace **(C)** and the peak of the release rate **(D)**. Red dotted line mark the moments 0.75 ms after action potentials, which roughly coincide with the peaks of release rate indicating a constant transmission delay.

We need to extract *q*(*t*) given *I*_syn_(*t*). Since *q*(*t*) and *A* always come together as a product in Equation (17), we can only estimate *q*(*t*) up to a scaling factor *A*. Because the asynchronous release events are relatively sparse, classical approaches, such as Fourier methods, are not suitable in estimating the release rate. In the Section 4, we thus develop an effective deconvolution method to estimate *Aq*(*t*) from *I*_syn_ (see Section 4.3, for details), and take care that the measured *I*_syn_ was not confounded by a leaky current (see Section 4.2). Figure 3 presents an example of the estimated release rate extracted from IPSC of the postsynaptic neuron. The reconstructed postsynaptic current based on the estimated release rate agrees well with the experimental data showing the adequateness of our deconvolution method.

#### 2.2.2. Estimating the parameters by probabilistic inference

Before fitting the SAR model, by comparing the membrane current at the presynaptic neuron with the extracted release rate, we observed that the peak of the release rate profile lags behind the peak of the membrane current by roughly a constant time 0.75 ms (Figures 3C,D). This implies that the transmission delay *D* = 0.75 in Equation (16).

We found further that the release rate increased sharply after 0.3 ms of the presynaptic spike, and decreased abruptly after 1.1 ms of the sharp increase. Thus, we divided the time trails into two types of time intervals: The first type started at 0.3 ms after each spike and lasted 1.1 ms, and we called these intervals the synchronous release periods $P_{\text{sr}}^{\left(k,i\right)}$, where *k* is the spike number and *i* the trial number. The second type was the time interval between two synchronous release periods and named asynchronous release periods $P_{\text{ar}}^{\left(k,i\right)}$. The integrated neurotransmitter release in the defined time periods is calculated as (with *r* = sr, ar)

(18) $$\begin{array}{l} M_{r}^{\left(k,i\right)}=\int_{t\in P_{r}^{\left(k,i\right)}}Aq(t)\text{ d}t. \end{array}$$

After calculating the integrated neurotransmitter release in each synchronous or asynchronous periods, we adopted a probabilistic inference method to estimate the parameters in the SAR model similar to Costa et al. (2013). Similar to the assumptions in Costa et al. (2013), we assume that the released neurotransmitters in a certain release period conforms to a Gaussian distribution. Therefore, we use only the sample mean and standard deviation for each period over trials *i*, i.e., $\mu_{r}^{\left(k\right)}=\underset{i}{\sum }M_{\text{r}}^{\left(k,i\right)}/n$ and $\sigma_{r}^{\left(k\right)}=\sqrt{\underset{i}{\sum }{\left(M_{\text{r}}^{\left(k,i\right)}-\mu_{r}^{\left(k\right)}\right)}^{2}/n}$, for fitting the model.

When **M_r_** collects the values for the released neurotransmitters in predefined intervals from experimental data, the likelihood that the parameter set **θ** = {τ_sr_, *U*_sr_, τ_ar_, *U*_ar_, τ_*d*_, *U*_max_} can match the experimental observations **M_r_** is *P*(**M_r_**|**θ**). Given each parameter set **θ** the log likelihood is

(19) $$\begin{array}{l} \log P\left(M_{r}|\text{θ}\right)=\sum_{r\in \{\text{sr},\text{ar}\}}\sum_{k=1}^{N_{sp}}\left(-\frac{{\left({\tilde{M}}_{r}^{\left(k\right)}-\mu_{r}^{\left(k\right)}\right)}^{2}}{2{\left(\sigma_{r}^{\left(k\right)}\right)}^{2}}-\log\sqrt{2\pi }\sigma_{r}^{\left(k\right)}\right), \end{array}$$

where ${\tilde{M}}_{r}^{\left(k\right)}$ are the amount of released neurotransmitters predicted by the SAR model in the corresponding periods for a given parameter set. The technical details of the fitting process are described in the Section 4.4.

**Figure 4:**
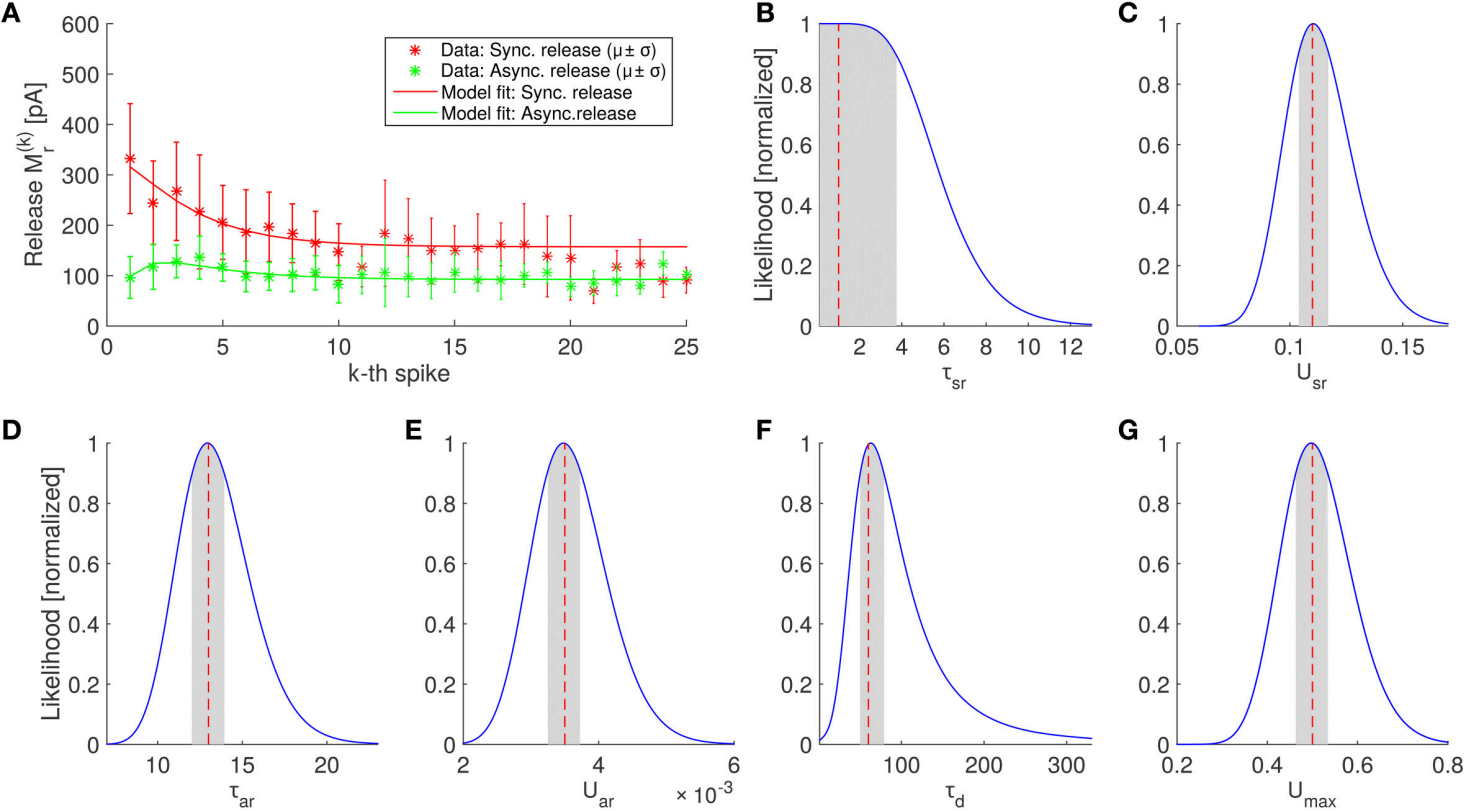
**Parameter estimation of the SAR model and comparison of the best fitting SAR model with experimental data**. **(A)** Average amount of neurotransmitter release in the periods after the *k*th spike in experiments (asterisks) for synchronous and asynchronous release periods (red and green colors, respectively) and prediction of the SAR model (solid lines). The model captures the experimental observation very well (log likelihood value –52.8259, see Equation 19), although the fit was only constraint by the first 10 spikes (see Section 4). Error bars indicate standard deviations. Note that the number of trails for each spike is different. In particular, for spikes 21–25 only 3 trials were available (see Section 4). **(B–G)** Scaled likelihood along each dimension around the optimal parameter set for the 6 fitted parameters of the SAR model. Shaded areas indicate the region where the likelihood is above 90% of the maximal value, when the single parameter is varied while all other remain fixed.

**Figure 5:**
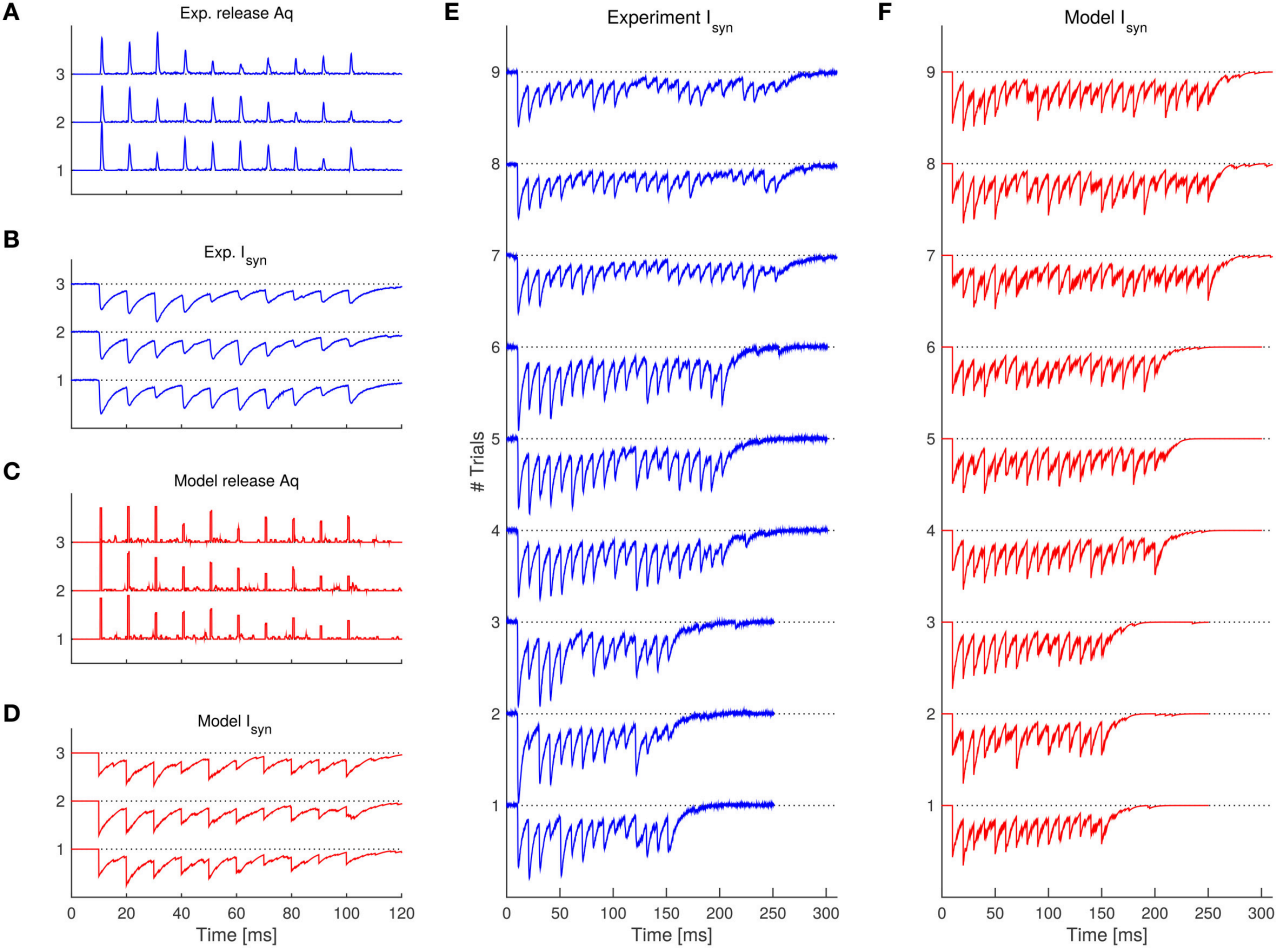
**Comparison of the experimental traces with the SAR model after fitting the parameters. (A)** Experimentally observed release rate for the 3 trials having 10 spikes. **(B)** Experimentally observed synaptic current corresponding to the release rate in **(A)**. **(C,D)** 3 example trials simulated with the SAR model with the same spike pattern as in **(A,B)**. Note that simulated traces are qualitatively similar with the experimental data. **(E,F)** Synaptic current comparison of the rest of the experimental trials **(E)** and further example realizations of the SAR model **(F)**. See Table 1 for parameters used. For **(A,C)**, a boxcar filter with 1 ms width was used to better visualize the instantaneous release events.

The results on the experimental data are shown in Table 1 and Figure 4. We found that the SAR model qualitatively captured the randomness and the general appearance of the experimental data (see Figure 5, for a comparison).

**Table 1:** **Fitted parameters that maximizes the log likelihood within the indicated search space using grid search for a FS-PC synapse**.

**Parameter**	**Unit**	**Value**	**Confidence interval**	**Search space**
*U*_sr_		0.11	[0.104, 0.117]	[0.02, 0.3]
τ_sr_	ms	1	[0.10, 3.7]	[1, 4]
*U*_ar_		0.0035	[0.00325, 0.0037]	[0.004, 0.024]/*U*_max_
τ_ar_	ms	13	[12, 14]	[4, 32]
τ_*d*_	ms	60	[51, 76]	[20, 80]
*U*_max_	1/ms	0.5	[0.06, >2]	[0.125, 2]
*N*_*F*_		271	n.a.	n.a.

After having estimated a suitable parameter set, we explore in the following effects of selected parameter variations on the response of the SAR model.

### 2.3. Effect on asynchronous release caused by slower Ca^2+^ buffering

For a fast-spiking interneuron of the epileptic human tissues, the estimated decay time constant of the asynchronous release was τ_ar_ = 8 ms (see Table 1), which means that the asynchronous release rate decays very fast after spiking activity. In cholecystokinin (CCK)-expressing interneurons, on the other hand, the post spike train asynchronous release was found to decay slower (Hefft and Jonas, 2005). Mechanistically, the fast decay of the release might be caused by the parvalbumin (PV) contained in the fast-spiking interneuron. Parvalbumin is a Ca^2+^-binding albumin protein, which can cause free Ca^2+^ concentration to rapidly decay causing a fast deactivation of Ca^2+^ sensors. Therefore, τ_ar_ in PV-containing fast-spiking interneuron synapses is expected to be relatively small. Accordingly, a previous study (Jiang et al., 2015) suggested that faster clearance of free Ca^2+^ could contribute to the reduction of the asynchronous release.

In experiments, the speed of free Ca^2+^ clearance can be controlled by the administration of the drug EGTA-AM, which enhances the speed of Ca^2+^ clearance (Otsu et al., 2004; Jiang et al., 2012). This would correspond to smaller τ_sr_ and τ_ar_ time constants in the SAR model. Because the two Ca^2+^ sensors have different biochemical properties, the influence of a slower decay of the free Ca^2+^ concentration on τ_sr_ and τ_ar_ may be different.

We ran the model with different τ_ar_ to see how the time decay of the Ca^2+^ concentration can affect asynchronous and synchronous release. For better illustration, we plot both releases separately. Specifically, based on the receptor model Equation (17), we can separate IPSCs in *I*_ar_(*t*) and *I*_sr_(*t*) according to

(20) $$\begin{array}{lll} I_{\text{syn}}\left(t\right) & =I_{\text{sr}}\left(t\right)+I_{\text{ar}}\left(t\right),\; & \; \end{array}$$

(21) $$\begin{array}{lll} I_{\text{ar}}\left(t\right) & =\int_{-\infty }^{t}e^{-\frac{t-t\prime }{\tau_{\text{GABA}}}}w\left(v-E_{\text{GABA}}\right)q_{\text{ar}}\left(t\prime \right)dt\prime ,\; & \; \end{array}$$

(22) $$\begin{array}{lll} I_{\text{sr}}\left(t\right) & =\int_{-\infty }^{t}e^{-\frac{t-t\prime }{\tau_{\text{GABA}}}}w\left(v-E_{\text{GABA}}\right)q_{\text{sr}}\left(t\prime \right)dt\prime .\; & \; \end{array}$$

**Figure 6:**
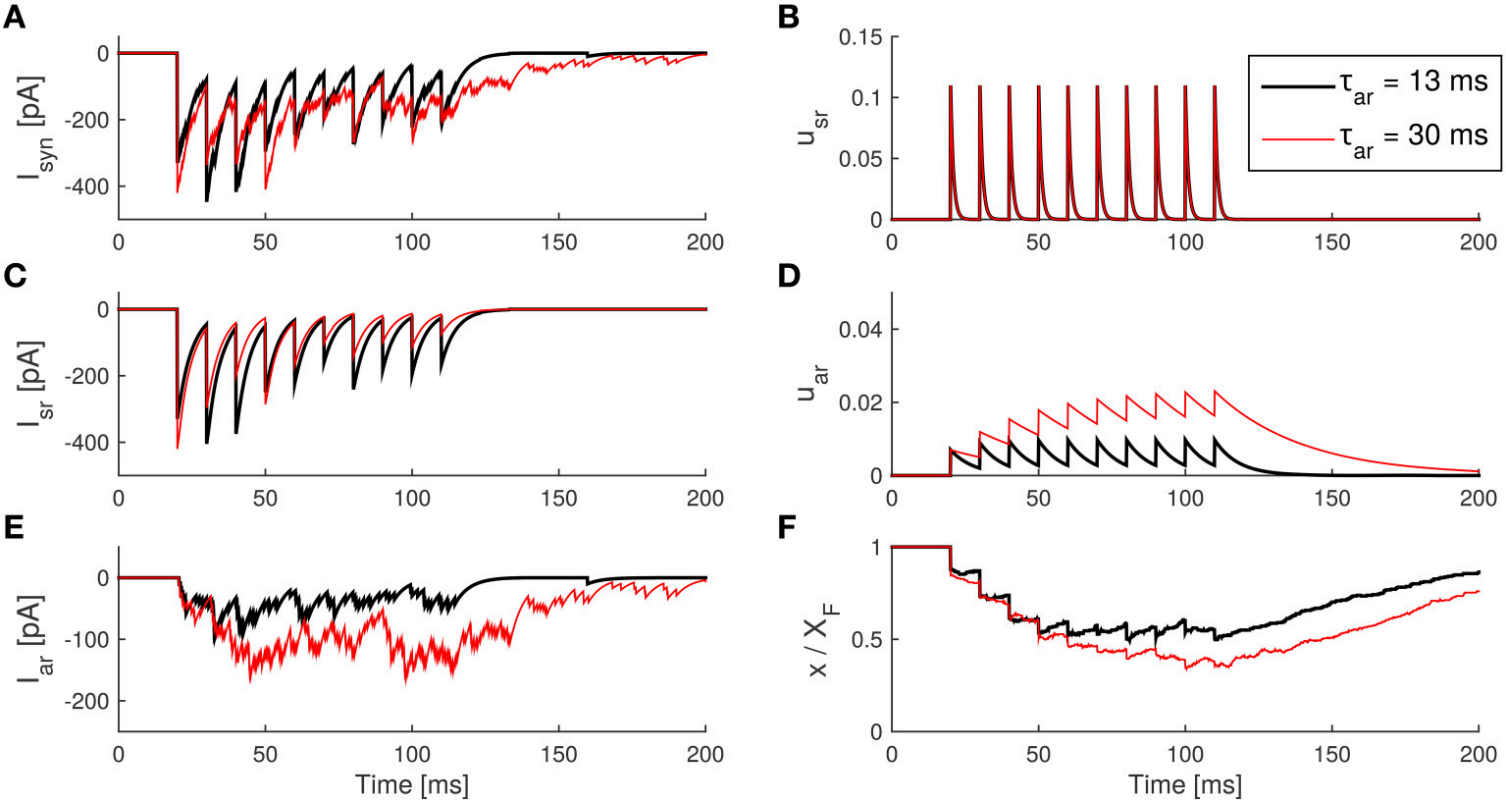
**Effect of an increase in the asynchronous release decay time constant τ_**ar**_ in the SAR model**. **(A)** The IPSCs caused by transmitter release. **(B)** The synchronous component of the IPSC caused by synchronous release. **(C)** The asynchronous IPSC caused by asynchronous release. **(D)** The internal state *u*_sr_ of the synapse model during the spike train. **(E)** The internal state *u*_ar_ during the spike train. **(F)** The internal state *x* (with a scale of 1:*X*_*F*_) during the spike train. *Parameters:* taken from Table 1 except τ_ar_ as indicated. The amplitude of the IPSC caused by a quantum release is *Ax*_0_ = 10 pA.

Increasing the time constant τ_ar_ from e.g., 8 to 30 ms made the asynchronous release rate *u*_ar_ visibly slower, so that *u*_ar_ accumulates and saturates at higher levels after repetitive spikes, in turn leading to a larger *I*_ar_ (see Figure 6). Moreover, the amount of free vesicles *x* decreases to lower levels as a result of the enhancement of asynchronous release, which also causes a smaller *I*_sr_ due to the resource competition of the two release processes. This form of competition was found in experimental data as well (Otsu et al., 2004). Finally, after the spike train, the asynchronous current lasts longer because τ_ar_ decays more slowly.

### 2.4. Effect on asynchronous release caused by stronger influx of Ca^2+^

In rat epileptic tissues, it was found that action potentials have higher amplitude than that in normal rats (Jiang et al., 2012). The higher amplitude could in principle lead to an increased Ca^2+^ influx, which will cause the Ca^2+^ sensors to be more active. Interestingly, it was found that manipulating the amplitude of action potentials had greater influence on the asynchronous release than on the synchronous release process (Jiang et al., 2012). The change of Ca^2+^ influx would correspond to an increase of *U*_sr_ and *U*_ar_ in the SAR model.

**Figure 7:**
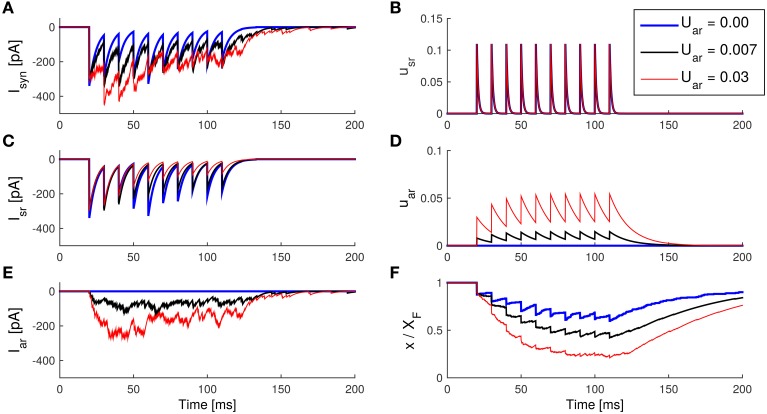
**Enhancement of asynchronous release with an increase of ***U***_**ar**_ in the SAR model**. **(A)** The IPSCs caused by total transmitter release. **(B)** The synchronous component of the IPSC caused by synchronous release. **(C)** The asynchronous IPSC caused by asynchronous release. **(D)** The internal state *u*_sr_ of the synapse model during the spike train. **(E)** The internal state *U*_ar_ during the spike train. **(F)** The internal state *x* (with a scale of 1:*X*_*F*_) during the spike train. Note that asynchronous release causes a decrease in the synchronous release due to the competition of vesicle resources. *Parameters:* taken from Table 1, except *U*_ar_ as indicated. *Ax*_0_ = 10 pA.

We therefore run the model with different *U*_ar_ to see the effects on *I*_sr_ and *I*_ar_ (results are shown in Figure 7). Increasing the parameter *U*_ar_ caused *u*_ar_ to rise to higher levels after each spike. Since τ_ar_ was short, *u*_ar_ saturated soon after a few spikes. Higher *u*_ar_ levels resulted in stronger asynchronous IPSC. Similar to the previous simulation, stronger asynchronous release depletes resources (that is, a decrease in *x*), and thus forces smaller synchronous IPSCs.

It is known that changes of Na^+^, K^+^, and Ca^2+^ channels can all contribute to epilepsy (Lytton, 2008). Changes in the channel dynamics might deform the waveform of action potentials, and thus in turn might lead to the enhancement of asynchronous release. In some experiments (Zengel and Magleby, 1977; Otsu et al., 2004), Ba^2+^, Sr^2+^, and Li^+^ are used to selectively enhance synchronous or asynchronous release. These changes can be modeled with the change of *U*_ar_ in the SAR model as described.

Although both, larger *U*_ar_ and larger τ_ar_, can enhance asynchronous release, there are some crucial differences on the release characteristics. With large τ_ar_, the asynchronous release rate *u*_ar_ will increase slowly during repetitive spikes, and then decay slowly after the spikes have ceased. Thus, *I*_ar_ does not increase much during the first few spikes, but lasts longer after the spike train. On the other hand, with large *U*_ar_, asynchronous release level *u*_ar_ increases rapidly after the first few spikes, and also decays fast after the spikes have ceased. Thus, in contrast to the case when varying the time constant, when changing *U*_ar_ the dynamics of *I*_ar_ is faster, while the level of asynchronous release during the spike train is elevated in both cases.

### 2.5. Quantum size controls the transmitter release variability

We found that the quantum size *x*_0_ mainly affects the variability of the asynchronous release.

Assume that the overall amount of released neurotransmitter stayed the same but each vesicles contained less neurotransmitter (implicating a increased numbers of vesicles in the active zone). This can be modeled by holding the synapse strength *wX*_*F*_ constant, when increasing the total available vesicles $\frac{X_{F}}{x_{0}}$ by reducing the released quantum *x*_0_. Note that *w* is the synaptic weight in the receptor model (see Equation 16).

**Figure 8:**
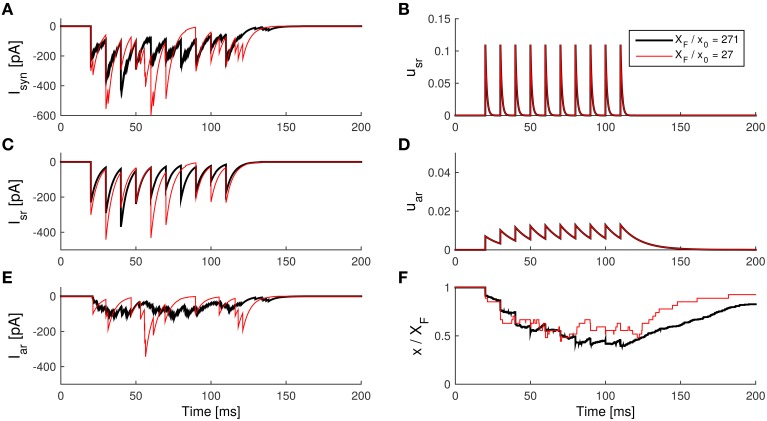
**Influence of the effective quantum size on the asynchronous release in the SAR model. (A)** The IPSCs caused by the total transmitter release. **(B)** The synchronous component of the IPSC caused by synchronous release. **(C)** The asynchronous component of the IPSC caused by asynchronous release. **(D)** The internal state *u*_sr_ of the synapse model during the spike train. **(E)** The internal state *U*_ar_ during the spike train. **(F)** The internal state *x* (with a scale of 1:*X*_*F*_) during the spike train. Note that the current caused by asynchronous release is visibly smoother if the released quantum size is smaller. *Parameters:* taken from Table 1, except *N*_*F*_ = *X*_*F*_/*x*_0_ as indicated. *Ax*_0_ = 10 pA.

Suppose that at a time *t*, the fraction of remaining neurotransmitters is ξ. Then there are $\frac{\xi X_{F}}{x_{0}}$ vesicles. If each vesicle had a release probability of *p* in a short period, then the variance of released vesicles is $\frac{\xi X_{F}}{x_{0}}p\left(1-p\right)$ based on the binomial release hypothesis. So the variance of the conductance *g* from Equation (16) is

(23) $$\begin{array}{l} \begin{array}{cc} {\left(wx_{0}\right)}^{2}\frac{\xi X_{F}}{x_{0}}p\left(1-p\right) & =wx_{0}\left(\xi wX_{F}\right)p\left(1-p\right) \end{array}\\ =wx_{0}c, \end{array}$$

where *c* is a constant, since *wX*_*F*_ is held constant. Thus, for larger *X*_*F*_/*x*_0_, *wx*_0_ becomes smaller, so that the variance of the conductance caused by neurotransmitter release becomes smaller. Therefore, smaller *wx*_0_ makes the IPSC generated by the asynchronous release appear smoother (see Figure 8).

## 3. Discussion

In this article, we developed a phenomenological synaptic model, the SAR model, that includes a description of the dynamics of both, asynchronous and synchronous neurotransmitter release. We based the derivation of the SAR model on a popular model for short-term plasticity, so that possible short-term synaptic plasticity dynamics of the synchronous release process can be described in addition to the asynchronous release dynamics. We found that our model agrees well with data on asynchronous release characteristics from experiments (Jiang et al., 2012) and in particular reproduced the effect of selectively enhancing or reducing asynchronous release found by experimental manipulation of the Ca^2+^-dynamics (Otsu et al., 2004).

### 3.1. Features of the model

A previous experimental study suggested that the asynchronous release phenomenon may be caused by two different release sites having different distances to the Ca^2+^ channels (Hefft and Jonas, 2005). However, this phenomenon can also be explained by only having one release site but different Ca^2+^ sensors that have distinct association, dissociation, and cooperativity properties. In our model, we thus assumed two different Ca^2+^ sensors for the two release processes. This could be a simplification, because in a much more biologically detailed model that only includes one kind of Ca^2+^ sensor but assumes a detailed vesicle life cycle and Ca^2+^ concentration dynamics, asynchronous release phenomenon also emerged (Pan and Zucker, 2009). However, for the purpose of building a simple phenomenological model, the assumption of having two pools of sensors competing for a common pool of free vesicles seems to be an adequate simplification and able to reproduce experimental data.

Another model was proposed for describing asynchronous release on a molecular basis using 3D Monte-Carlo simulations of Ca^2+^ diffusion in a single synapse (Nadkarni et al., 2010). However, these molecular details for describing the asynchronous release process in the hippocampal synapse makes the model very computationally expensive and thus unsuitable for large scale network simulations. Computational efficiency in simulating synaptic interactions is in particular important because in a network of *N* neurons, the number of synapses is proportional to *N*^2^. Thus, the dynamics of synapses are the most costly aspect to compute in typical neural network simulations. When considered in addition to the synchronous release process already present in a network simulation, the asynchronous release in our SAR model adds only a single dynamical equation (for the asynchronous release rate) together with two noise processes (one for replenishment, one for the released amount) per synapse and time step for simulating the dynamics of a neural network.

Our model is thus simple and computationally efficient while still catching some key features of the asynchronous release found in the experimental studies. Note that also the STP model, which we use for describing the synchronous release process in the SAR model, is in itself only an phenomenologically approximation to the complex biophysical mechanism of synchronous release (Tsodyks and Wu, 2013). Taken together, the SAR model is thus suitable to be used in large-scale network simulation to investigate the effect of asynchronous release on network dynamics. Parameters of the SAR model can be adjusted to adapt for different contributions of synchronous and asynchronous release as illustrated in the Section 2.

### 3.2. Possible extension of the model

Our model does not include a positive stationary probability of asynchronous release in the absence of spike, because the release probability rate will decay to zero (with time constant τ_ar_) when spiking ceases (compare to Equation 7). However, it is known that in some synapses, spontaneous release events occur even in the absent of spikes (Kavalali, 2015). We here neglected spontaneous release, since in our data single events are tiny and thus cannot be reliably distinguished from background noise. However, if needed, this rate could be easily incorporated in the SAR model. In Equation (7), the steady state had to be changed to some positive spontaneous rate, e.g., *U*_0_, that is the equation would be modified to

(24) $$\begin{array}{l} \frac{du_{\text{ar}}\left(t\right)}{dt}=-\frac{u_{\text{ar}}\left(t\right)-U_{0}}{\tau_{\text{ar}}}+U_{\text{ar}}\left[U_{\text{max}}-u_{\text{ar}}\left(t\right)\right]\underset{m}{\sum }\delta \left(t-t_{m}\right). \end{array}$$

This would yield spontaneous release events in the absent of spiking activity in the SAR model.

### 3.3. Release rate estimation from experimental trials

Some assumptions during the estimation of the model parameters may not hold exactly in the reality, possible confounding the accuracy of the estimated release rate from experimental trails. For example, it was assumed that the leaky current was constant, but in fact, it may change with the membrane voltage and the internal state of the neuron. Moreover, the reversal potential *E*_*GABA*_ was also assumed to be constant. However, in high frequency spike trains it is known that a neuron may not be able to maintain ion concentration stable (Staley et al., 1995), which may lead to a drift in *E*_*GABA*_.

In the estimation of the parameters of the SAR model, the receptor dynamics was assumed to be a simple exponential decay process. This assumptions might be too restrictive for some synapse types, for instance, because any receptor saturation is neglected. While the fitting process might be more difficult with more complex receptor dynamics, the core of the SAR model is still valid as it models the neurotransmitter release process and not the receptor dynamics.

Taken together, there are some assumptions that limit the precision of the estimation of the release rate from IPSC, which in turn might affect the accuracy of the parameter estimation of the SAR model. Although quantitative aspects might slightly change, it is unlikely that the above assumptions would compromise a useful qualitative description of the asynchronous release process by the SAR model.

### 3.4. Categorizing the synchronous and asynchronous release periods from the experimental observed IPSC

Though synchronous and asynchronous release have different underlying mechanisms, it is difficult to distinguish them from the postsynaptic currents alone. Some experimental studies (Pan and Zucker, 2009; Wen et al., 2010) chose the neuromuscular junctions as model system because the receptor's conductance decay constant is very short. It is thus easy to directly identify both release processes from the measured synaptic currents. This method gives both the timing and relative amount of neurotransmitter release, but it is limited to certain kinds of synapses. Other studies (Jiang et al., 2012, 2015) used the slope of the IPSC after the spike train to identify events of asynchronous release. This method can give the timing of asynchronous release, but cannot give the amount of released neurotransmitters at the same time.

If the receptor decay time constant is not short enough, the asynchronous IPSC will be mingled together with the synchronous IPSC, and it is not easy to categorize the release as synchronous or asynchronous. In this article in contrast to previous method, we thus developed a deconvolution approach to account for the finite conductance decay time constant to directly get an estimation of the transmitter release rate. We then used the timing of the release rate (and not that of the IPSCs) to achieve categorization since it is more reasonable to assume that synchronous release rate can be described by a instantaneous pulse functions.

### 3.5. Applicability to other synapse types

To fit the SAR model we used data obtained from a synapse of a fast spiking interneuron to pyramidal cell. We found that the average release rate changes after spikes were well captured by the model yielding simulations that were qualitative comparable to the experimental traces. Note that the model is also suitable for modeling synapses with negligible asynchronous release, such as PC-PC synapses, since the SAR model generalizes the established STP model of the synchronous release. Data were too limited to more quantitatively test how well other more detailed aspects, i.e., the form of the distribution of asynchronous events, were predicted after fitting the model. Since we assumed only very simple release dynamics, the SAR model can only be seen as a first approximation to the underlying biophysics. The variability of the parameters of the model for other synapses of the same type or between different synapse types is not addressed here and needs future experimental studies, where the SAR model and our methods could be used to fit data from many synapses of the same type (but from different neurons or areas) and between different types of synapses.

### 3.6. Conclusion

We here presented a new model for the phenomenological description of synchronous and asynchronous neurotransmitter release at chemical synapses. Since our model extends a well-known STP model often used in large-scale network simulations, it should be easy to include it in future modeling studies. We hope that the SAR model will facilitate the understanding of the computational properties and neural function of the asynchronous release process.

## 4. Materials and methods

### 4.1. Experimental methods

The experimental data used for the fitting of the SAR model was taken from a previous study (Jiang et al., 2012). We thus refer to Jiang et al. (2012) for a detailed description of the experimental protocol. In brief, human neocortical tissues obtained from patients with intractable epilepsy were sectioned in ice-cold sucrose-based slicing solution, and then maintained in artificial cerebrospinal fluid at 35.5C°. Whole-cell recordings were performed in synaptically connected layer 5 fast-spiking and pyramidal cell pairs (FS-PC) with high-chloride internal solution. The presynaptic FS cells were stimulated by current pulses to generate action potentials. Asynchronous release (AR) events could be detected in the postsynaptic PC during and after the action potentials.

### 4.2. Estimating the synaptic current

The leaky current should be subtracted from the recorded current, so that the remaining part is purely the synaptic current *I*_syn_(*t*) caused by neurotransmitter release. Here, we estimate the leaky current *I*_leaky_ to be the mean of the recorded current in a short period before each spike train. In principle, *I*_syn_(*t*) should be non-positive for inhibitory synapses. However, in some trials, there were some time points where *I*_syn_(*t*) > 0, probably due to small noise fluctuations. We thus set *I*_syn_(*t*) to a small negative value −ϵ where *I*_syn_(*t*) >−ϵ. Here ϵ should be small enough so that it does not introduce much bias compared to the amount of noise. We set ϵ to 0.2 pA, which is much smaller than the amplitude of the IPSC.

### 4.3. The deconvolution method

We found that classical approaches to deconvolution are ineffective for inferring the release events. We thus develop in the following a new deconvolution approach.

Consider that a time course of a postsynaptic current *I*_syn_(*t*) in a time interval [0, *T*] with a temporal resolution d*t* = 0.05 ms was recorded. After pre-processing (see Section 4.2), we divided the time interval into a sequence of *N* = *T*/d*t* time bins with size *dt*. In discrete form, the synaptic current in the *i*th time bin according to the model is given by (compare to Equation 17),

(25) $$\begin{array}{l} I_{\text{syn}}(i)=\sum_{j=1}^{i}K(i-j)q(j-\Delta )\text{ d}t, \end{array}$$

where the kernel is $K\left(i-j\right)=Ae^{-\left(i-j\right)dt/\tau_{\text{GABA}}}$ when *i* ≥ *j* and otherwise 0 and Δ = *D*/d*t* is the transmission delay.

First, we estimate the GABA receptor's decay time constant τ_GABA_ by estimating the kernel *K* from the data using MATLAB's curve fitting toolbox.

**Figure 9:**
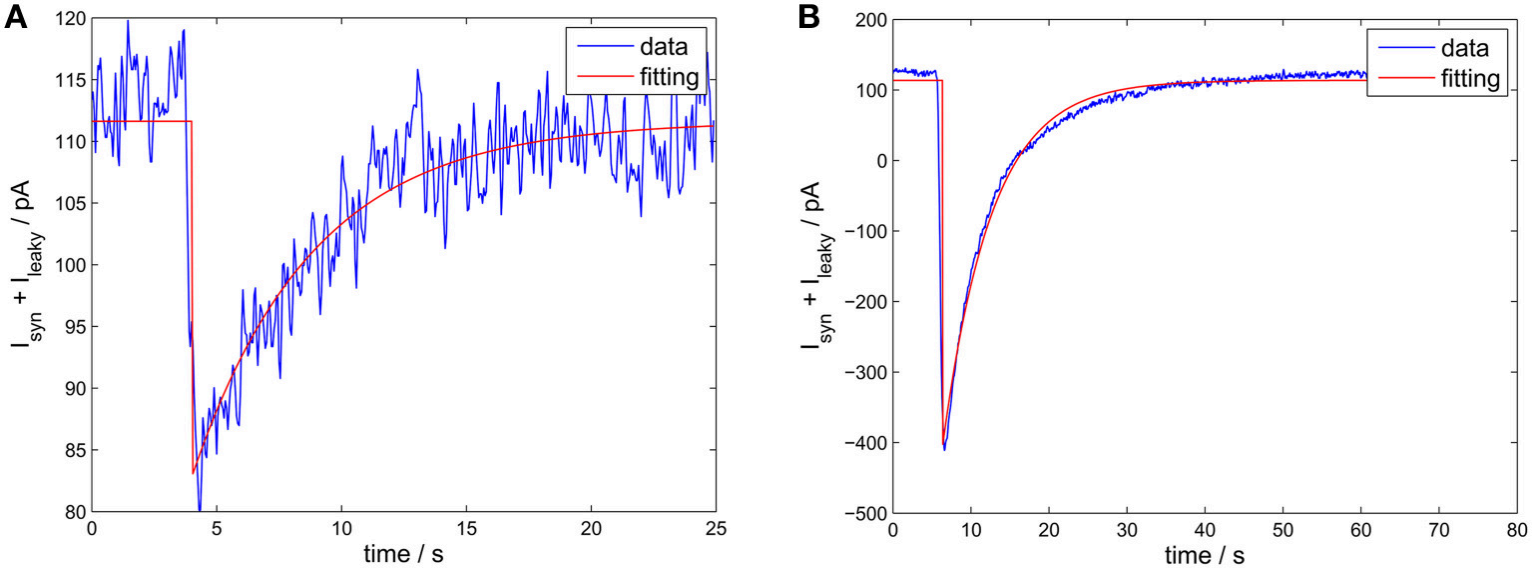
**Estimating the receptor decay time constant**. The experimental data is from a synapse of epileptic human tissue connecting a fast-spiking interneuron with a pyramidal neuron. Blue curve: the IPSCs. Red curve: the fitted IPSC using an exponential decay function (with offset). **(A)** A spontaneous release event. The estimated τ_GABA_ was 4.8 ms (confidence interval 4.39–5.27 ms). **(B)** Single action potential triggered release. The estimated τ_GABA_ was 6.2 ms (confidence interval 6.06–6.46 ms).

The time constant τ_GABA_ could be estimated from spontaneous neurotransmitter release events or from single action potentials. However, both estimates were similar (see Figure 9). For a synapse between fast-spiking neurons, we found τ_GABA_ ≈ 2.6 ms, for a synapse from fast-spiking neurons to pyramidal neurons, τ_GABA_ ≈ 5 ms, which is in good agreement with values reported in the literature (Bartos et al., 2002).

Having estimated the form of the kernel, we adopted a sequential approach to estimate *q*(*i* − Δ) one-by-one, starting from *i* = 1 to *i* = *N*. We start with *q*(1 − Δ) = 0. At the *i*th step, we assume that the *q*(*j* − Δ) for steps *j* = 1, …, *i* − 1 are already estimated. We then choose the *q*(*i* − Δ) by satisfying

(26) $$\begin{array}{l} q(i-\Delta )=\underset{p\geq 0}{\arg\min}|\sum_{j=1}^{i-1}K(i-j)q(j-\Delta )\text{d}t+Ap\text{ d}t-{\tilde{I}}_{\text{syn}}(i)|\\ \;\equiv \underset{p\geq 0}{\arg\min}|J(i)|,\\ \text{subject to }{\tilde{I}}_{\text{syn}}(k)\geq e^{-(k-i)\text{d}t/\tau_{\text{GABA}}}J(i)\;\forall k\geq i, \end{array}$$

where Ĩ_syn_(*i*) is the experimentally measured IPSC in the time interval *i* and *J*(*i*) is an abbreviation of the term to maximize. Note that the optimization constraints achieves that the release rate at time *t* can only grow if the future decay can be accommodated. This procedure resulted in an good estimate of the release rate (see Figure 3, for an example).

### 4.4. Parameter fitting

We used *n* = 12 trials of release rate under spike frequency of 100 Hz and spike number of 10 (3 trials), 15 (3 trials), 20 (3 trials), and 25 (3 trials). For comparability, only the release rate statistics of first *N*_*sp*_ = 10 spikes were used for all trails. Incorporating more spikes did not improve the fit, because (a) the usable trials number was reduced and (b) the release rate statistic change started to saturate after about 10 spikes in this data set. Because the amount of available trails were limited, we divided the spike trails in short intervals and optimized the parameters of the model to match the average estimated neurotransmitter within these short intervals. We inferred the model parameters by minimizing the log likelihood given in Equation (19) of the main text.

Since the amount of released neurotransmitters in the SAR model is stochastic, the released amount ${\tilde{M}}_{r}^{\left(k\right)}$ in each of the time periods defined in the main text should be averaged over trials. Since we only fit the mean of the experimental data, to save time in calculating ${\tilde{M}}_{r}^{\left(k\right)}$ for purpose of fitting, we modified the Equation (9) of the SAR model to

(27) $$\begin{array}{l} q_{\text{ar}}(t)\text{ d}t=x(t)u_{\text{ar}}(t)\text{ d}t, \end{array}$$

and additionally used the deterministic model of the synchronous release described in the main text, so that there is no stochasticity in the model during fitting. Calculating mean release rate in this way was much faster than taking the average of release rate over many trials generated by the model in Section 2.1.2 but does not change the results. This modification also makes *x*_0_ a meaningless parameter during the fitting. We thus estimated quantum size *x*_0_ later using another method (see Section 4.4.2 below).

After we calculated the generated release rate *q*′(*t*) by the model, we computed the model response ${\tilde{M}}_{r}^{\left(k\right)}$ as described for the experimental data [the synchronous release periods $P_{\text{sr}}^{\left(k,i\right)}$ started immediately when there was synchronous release and also lasted for 1.1 ms], except that we accounted for the normalization factor *A* [corresponding to *w*(*v* − *E*_*GABA*_)] to ensure that the sum $\sum {\tilde{M}}_{r}^{\left(k\right)}$ corresponded to that of the experimental data.

Note that the released neurotransmitters increase proportionally to the parameter *X*_*F*_ (see Equations 6, 7, 10, and 2), so that $A\propto \frac{1}{X_{F}}$. This means that *X*_*F*_ is not constrained if *w*(*v*−*E*_*GABA*_) is not known. Thus, we arbitrarily set *X*_*F*_ = 1 in the fitting process.

A grid search was used to find the parameter set which maximized the log likelihood. See Table 1 in the main text for the grid space used and the inferred parameters.

After finding the optimal parameter set $\hat{\theta }$, we examined the nearby space around it with thinner bin size and smaller range along each parameter dimension to estimate a confidence region. The confidence interval along the each dimension was taken where the likelihood was above 90% of the maximal likelihood value. See Table 1, for the list of the determined confidence intervals.

#### 4.4.1. Validating the fitting method on simulated data

To check how reliably the fitting method finds the true parameters of the model, we tested it on artificially generated data with the SAR model. For that, we randomly chose 120 random parameter settings (in the ranges τ_sr_ ∈ [4, 10], *U*_sr_ ∈ [0.1, 0.5], τ_ar_ ∈ [8, 20], *U*_ar_ ∈ [0.004, 0.02], τ_*d*_ ∈ [20, 80], *U*_max_ ∈ [0.2, 1], and *N*_*F*_ = 271), simulated released neurotransmitter for spike trains with 25 action potentials (100 Hz) for 50 trials and performed grid search for all 6 parameter.

We found that when comparing the estimated with the true values, the parameters *U*_sr_ and τ_d_ could be very reliably estimated (*U*_sr_: *R*^2^ = 0.9261; τ_*d*_: *R*^2^ = 0.957). We thus fixed the values found for these two parameter to save computation time and performed a finer grid search for the other 4 free parameters. This yielded values very close to the true values for τ_ar_ (*R*^2^ = 0.81) and still reasonable values for τ_sr_ (*R*^2^ = 0.49). The reason why τ_sr_ could be less reliably estimated, is that for moderate spike rates and moderate facilitation, the exact time constant of the synchronous release rate does not affect the dynamics of the model significantly as long as it decays almost to zero within the inter-spike interval (see e.g., Figure 6B).

Finally, we found that although the overall quality of the fit to the simulated data was very good throughout (on average 1% relative deviation of the likelihood of the estimated parameter compared to the likelihood of the true model on the same data, averaged over all 120 groups of parameters), the estimated parameters *U*_max_ and *U*_ar_ were in general not close to the true values (*U*_max_: *R*^2^ = 0.25; *U*_ar_: *R*^2^ = 0.2). This could be understood by reviewing the SAR model formulation. Note that the parameter *U*_max_ is somewhat redundant with the parameter *U*_ar_, because they both increase the update of the asynchronous release rate (see Equation 7). Indeed, we found that the product *U*_max_*U*_ar_ was correctly estimated by our fitting method and very close to the true values (*R*^2^ = 0.93). In other words, for relatively short spike trains with moderate firing rate, the saturation level of the asynchronous release process, *U*_max_, is seldom reached, so that an incorrect setting of *U*_max_ does not significantly affect the dynamics as it can be compensated for by an appropriate setting of *U*_ar_. For our data, we found that fixing *U*_max_ to a constant number e.g., $U_{\text{max}}=1{\text{ms}}^{-1}$, and performing the grid search only on the remaining 5 parameters yielded good results in practice.

#### 4.4.2. Estimating the quantum size

After the parameters are found, one can calculate the quantity of *x*_0_. According to the receptor model, the amplitude of the IPSC triggered by a release of an individual quantum neurotransmitters is ${\tilde{x}}_{0}=A\cdot x_{0}$, with $x_{0}=\frac{{\tilde{x}}_{0}}{A}$ the quantum size. *A* is the normalization factor and ${\tilde{x}}_{0}$ can be estimated based on the statistics of the post train release rate as follows.

Similar to the case of fitting the release rate, we can only estimate ${\tilde{x}}_{0}=w\left(v-E_{GABA}\right)x_{0}=Ax_{0}$, that is the amplitude of IPSC generated by the release of a single vesicle.

For that, we collected the postsynaptic currents generated by pure asynchronous release, i.e., those after the synchronous release due to presynaptic action potentials are ceased (Jiang et al., 2012). We divided the time into small intervals of equal length with Δ*t* = 3.9 ms. At each interval *j*, the quantity $M\left(j\right)=\tilde{q}\left(j\right)\Delta t$ was extracted using the deconvolution method described in Section 4.3.

The vesicle fusion associated with asynchronous release is a binomial process, which can be approximated by a Poisson process. In particular, a previous study found that the mean and variance of post spike train IPSC are linearly correlated (Hefft and Jonas, 2005), which is an indicator for a Poisson process. Although the number of released vesicles is in general an inhomogeneous Poisson process over time, in a short interval, it can be treated as homogeneous. Thus, the ratio between its mean and variance (that is the Fano factor) should equal to 1 in each small interval. We use this property to infer the value of ${\tilde{x}}_{0}$.

**Figure 10:**
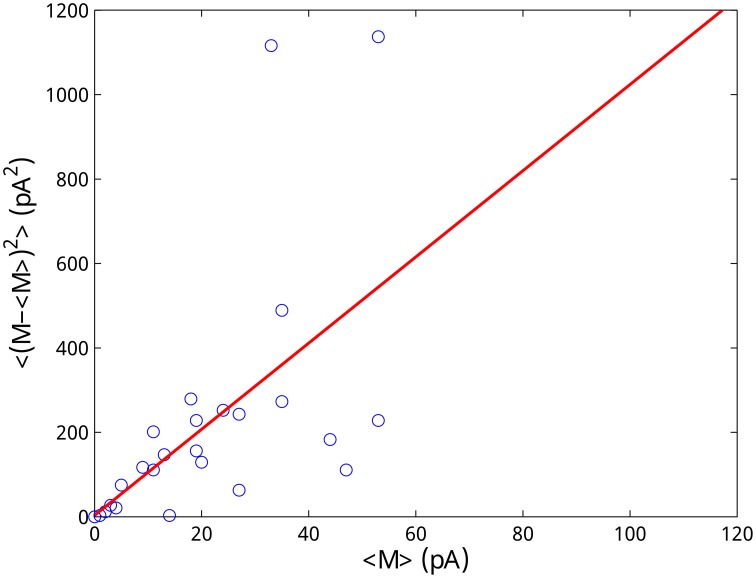
**Estimation of the amplitude of the postsynaptic current due to the release of a single vesicle**. The Poisson nature of the vesicle release process is utilized to estimate the quantum size. Each data point represents the mean and the variance of *M*(*j*) obtained from 6 trials. Linear regression (*R*^2^ = 0.89) gives an estimation of ${\tilde{x}}_{0}=10.21$ pA.

Denote *p*(*j*) a random number associated with the Poisson release at the *j*th interval of each trial. The amount of neurotransmitters released in this interval is thus given by $M\left(j\right)={\tilde{x}}_{0}p\left(j\right)$. For the Fano factor *F*{*M*(*j*)} for each interval *j*, that is the ratio between the mean and the variance of *M*(*j*) over trials, should thus hold

(28) $$\begin{array}{l} F\left\{M(j)\right\}={\tilde{x}}_{0}F\left\{p(j)\right\}={\tilde{x}}_{0}, \end{array}$$

because the Fano factor of a Poisson process is 1. By calculating the mean and variance of *M*(*j*), *j* = 1, …, *N*, from the experimental data and fitting them by linear regression, we obtained ${\tilde{x}}_{0}=10.21$ pA (see Figure 10). This value is in the range of the amplitude of the minimum spontaneous postsynaptic current (10–20 pA) recorded in the experiments (Jiang et al., 2012) validating our approach.

## Author contributions

Designed research: SW, YS, TW. Mathematical model formulation and simulation: TW, MR, SW. Analyzed data: TW, LY, XZ, MR, YS. Wrote paper: TW, MR, SW.

## Funding

This work was supported by the National Basic Research Program of China [2014CB846101 (SW, MR), and 2011CBA00400 (YS)], the National Natural Science Foundation of China [31371109 (MR), 31261160495 (SW), 31430038 (YS), and 31025012 (YS)], the Fundamental Research Funds for the Central Universities [248105584GK (SW)], and the Specialized Research Fund for the Doctoral Program of Higher Education [20130003110022 (SW)].

### Conflict of interest statement

The authors declare that the research was conducted in the absence of any commercial or financial relationships that could be construed as a potential conflict of interest.
